# What Shall We Do with Our Consumptives?

**Published:** 1902-04

**Authors:** C. H. Wilkinson

**Affiliations:** Galveston, Texas


For Texas Medical Journal.

### What Shall We Do With Our Consumptives?

BY C. H. WILKINSON, M. D., GALVESTON, TEXAS.

Any one who has investigated the subject of pulmonary consumption, as it prevails in the United States, must long ago have noted the fact that it by far outranks every other known enemy to human life in point of destructiveness, and that so far it has resisted the ingenuity of man in accomplishing its subjugation. It stands today the leading cause of death in nearly every city of our country, and in its ravages during the past century has destroyed more human lives than any pestilence or epidemic that has visited us in all that time.

This disease has prevailed uninterruptedly for the past one hundred years, and today is about as uncontrollable by ordinary means as it was a century ago.

Our country is teeming with it, although some sections are more crowded with it than others, and in the light of these reflections well may we ask ourselves the question, "What are we going to do with our consumptives?"

Another fact in connection with this subject is the unsatisfactory results usually attendant on our ordinary methods of dealing with tuberculosis. Remedy after remedy has had its trial, vaunted "cures" and specifics have proved unsuccessful, and but for the hope so characteristic of this class of invalids, three-fourths of all such remedies would never have enjoyed other than a brief existence. It is not intended, however, to decry remedial agéncies in the treatment of consumptives, since many of them are most valuable aids in producing comfort and prolonging life, but it is an undisputed fact that no system of medication so far discovered has proven a specific in the treatment of this disease. Men sicken, rally, and even seem to be entirely cured under many of these agencies, yet the majority of such cases die, and this compendium of its course is the usual result of every form of treatment practiced at the present time. In other words, there is today no medicinal agent or system of treatment that can be relied upon to cure consumption.

There are other facts in connection with this subject which might be dwelt upon, but I will only call to mind a single additional one of them before suggesting an answer to the question asked at the beginning of this article.

Despairing of hope in the usual methods employed, thousands of consumptives have turned their faces west in search of climate, and it is to this particular class of pulmonary cases that I would wish attention called to. The fame of Texas and New Mexico for the relief of phthisis has long since reached the shores of the Atlantic, and the response of invalids to this beckon of hygeia has gone up over every State and community in the Union. Thousands, as remarked above, have turned west to drink this mountain atmosphere, and today, as might be expected, we find our Western towns and cities, hills and valleys, swarming with this army of health seekers, each eager to regain that vitality which he rightfully thought he was entitled to.

In the rapid change from languor to health they have recognized the fact that at last the true remedy was discovered. Of such health seekers Texas has her quota, and thus from San Antonio to El Paso scarcely a village can be found without its "lunger."

And here the writer wishes to emphasize the fact that in West Texas we have, indeed, the natural sanitarium of the world: the climate is surpassed by none the sun shines on for healthfulness, and especially is it adapted to the cure of phthisis, and right well have they directed their footsteps who have gone there for the benefit of health.

In this particular section can be found phthisis in every stage. Cases well suited and those, on the other hand, most unsuited for such residence, are often seen indiscriminately associated, wandering from place to place, or oftener congregated at some boarding house or hotel, without guide or adviser, figuratively "at sea," without compass or rudder, and innocently infecting everything and every person around them with the germs of their disease.

While the climate they are enjoying is the best on earth, the conditions connected with their sojourn are illy calculated to hasten their recovery. It is of this class in particular that I would ask the question, "What are we going to do with them?" The writer during the past two years has had occasion to visit many of the places out West resorted to by consumptives, and he has been deeply impressed at what he saw. These resorts, as they are called, were usually characterized by the presence of numerous pale and attenuated young men and women, all there for the benefit of health, yet nearly every one of them living in open violation of some hygienic law. The consequences of drifting about without an intelligent friend or adviser, and living in utter disregard of many of the laws of sanitation, might easily be inferred.

Such cases either lengthened out the period of their cure, or else, wearied of waiting, and after leaving colonies of germs for others to inherit, prematurely hastened home to relapse and die.

In one city I visited recently, while passing along the street, I was called in to an undertaker's shop to see "a pretty piece of embalming." There lay a poor fellow dead with consumption, with a bullet hole through his brain, showing where his self-administered remedy had entered. He had been drifting about in search of relief from his disease, and while at an hotel, and discouraged at a hemorrhage, he took this effective method of ending his complaint.

This case so impressed me at the time that I determined to lay the matter before the profession, with the view of attracting their attention to the necessity of making some practical effort to furnish relief to this particular class of sufferers. Surely, with the present light of science and experience before us, and in view of the large number of these afflicted human beings, something more than the ordinary methods should prevail in attempting to banish this white plague from the face of the earth.

That it will be done eventually, however, I firmly believe, but when and how I am unable to foretell. In the meantime, while awaiting for this happy consummation, we should be up and doing in this direction. It is obligatory upon us to take up this important question (by far the most important one before the medical profession today), and if possible, work it to a finish.

Experience teaches us that none of the ordinary methods of treatment by medicine can be relied upon for the cure of consumptives. While many lives are undoubtedly prolonged, the vast majority of such cases perish by such methods, and thus it behooves us to turn away from our pharmacopœa in search of some reliable agency to cure our cases with. Fortunately, we have such agency right here at our door.

CLIMATIC TREATMENT.

In climate we have the cherished desideratum, experience having proved that when properly selected and properly taken advantage of its power for good can be relied upon.

Its benefits in phthisis are no longer a matter of speculation, but one of fact. In the presence of its magnificent healthful possibilities, all other remedial agencies pale into insignificance.

Climate, then, is the remedy *par excellence* to effect our cures, and right here in our own State, almost at our doors, in the elevated regions of West Texas, we have the most superb climate for the thorough and lasting cure of consumptive cases that can be found in the world.

Why? Because it possesses all the factors intended by nature to destroy the tubercular germ. Dryness, elevation, salubrity and the proper temperature for mostly the year round render Western Texas climate the superior of any to be found on the globe for the satisfactory treatment of consumptives.

It is in this atmosphere that raw meat suspended in the shade will remain fresh for weeks at a time. It is here that suppurative processes are rarely ever met in surgery.

West Texas can truly be styled the El Dorado for consumptives. Its dry and pure air, its elevation, ranging from 500 to 5000 feet above the level of the sea, and its scanty vegetation free from miasmatic germs, all combined, make it the ideal portion of the United States for consumptive health resorts.

Today there are thousands out there realizing the truth of these assertions, but unfortunately, many of these health seekers are improperly being cared for. They are practically left to shift for themselves, the smiling host at most of the resorts out there being satisfied to take their lucre and let nature do the balance.

These cases, usually so sensitive, above all things, need advice—advice of the proper kind; also encouragement, and even sympathy artfully administered. Besides these, healthful exercise in the sunshine and open air should be insisted on, hygienic environments should be maintained at all times, and proper food supplied. These requirements might be styled the "essentials" to the successful treatment of consumption. With them, in properly selected cases, 85 per cent. of all who sojourn in this country long enough will certainly be cured. Without them, the finest climate in the world will fail.

The field is a large one to operate in, and the material to work upon is abundant. The question is up to the medical profession, and we should act upon it without delay. Could this subject of our consumptives be clearly and fully laid before the eyes of some rich philanthropist the writer has no doubt but what this class would receive the aid their conditions appeal for, and in course of time the consumptive would outrank all other classes of unfortunates in the matter of sympathy and aid. In the meantime, while awaiting the action of the philanthropist, it devolves upon us to be doing something for them.

THE WRITER'S PLAN.

Bearing in mind the salient facts just enumerated in the make-up of this important subject, it should be easy for us to recognize our duty to the consumptive cases in our State.

Elsewhere I have dwelt upon this matter (TEXAS MEDICAL JOURNAL, November, 1901), but from the frequency and nature of the inquiries that have been received upon this question, I am satisfied that it has aroused public interest and requires further elucidation.

For the proper care of our consumptive cases we should establish and maintain proper resorts for them in Western Texas. As stated before, the climate there is all that sanitation could require, while the material to be cared for is already crowded on the ground. Build sanitaria, hotels, barracks, camps or anything constructed properly to shelter these cases, and guide and direct and manage them in such a manner at least that the invalid will perceive that an interest was being taken in his case and an effort was being made to bring him back to health.

Preferably I would suggest the hotel and camp resort combined, since in this combination it is believed that the maximum of comfort and success will follow.

Camp life for consumptives is now being practiced in Colorado and elsewhere, even in winter, and is proving a success. If cases thrive in Colorado camps in winter, much better can they do so here in Texas.

Purchase a plat of ground, forty acres for instance, watered and drainable; build thereon a small modern hotel, and pitch thereabouts your camp of tents. Establish rules of discipline and hygiene, and conduct your enterprise on a respectable and humane basis, and a reward will follow your efforts. Such an enterprise need not be expensive. Fifty thousand dollars would be sufficient, if judiciously expended, to build and furnish a small *modern* hotel, to purchase grounds and fifty tents, and to furnish the latter. There are many eligible places in West Texas where all this could be done for the amount named, and a dozen of such enterprises might be established.

There would be no lack of material to supply such places with, there would be no question of remuneration and success if conducted along the lines suggested. As a matter of course, a physician should be connected with such places to advise and direct the invalids.

As to raising the means for promoting so laudable an enterprise, there are several suggestions offered, but inasmuch as the measure is one that involves the treatment of invalids it properly should be championed by the medical profession. Not necessarily so, it is true, but preferably so, if it is possible.

Let the doctors of our State club together and build such sanitaria in different portions of Western Texas. We could easily maintain a dozen out there, since the country at large could be relied upon at all times in the near future to furnish the material to supply them with.

The writer believes that this plan of dealing with consumptives is today the only rational and encouraging, as surely it is the most humane method at our command, and it fully answers the question propounded at the outset of this article.

Should any person feel interested in pursuing this subject further, it would be a pleasure to enter into correspondence with him to that end.

---

For Texas Medical Journal.

